# Glyoxalase 1 is a proadipogenic gene

**DOI:** 10.1016/j.jbc.2025.110926

**Published:** 2025-11-07

**Authors:** Marissa N. Trujillo, Wei-Chen Zhang, Emely A. Hoffman, Naoya Kitamura, Aiden M. Phoebe, James J. Galligan

**Affiliations:** Department of Pharmacology and Toxicology, College of Pharmacy, University of Arizona, Tucson, Arizona, USA

**Keywords:** glyoxalase, GLO1, methylglyoxal, glycation, glycerol

## Abstract

Diabetes is one of the most prevalent and widespread diseases, with the majority of cases stemming from prolonged obesity. Obesity occurs through the expansion of adipose tissue in an unhealthy and dysfunctional manner, where patients develop inflammation and insulin resistance. Diabetic patients have increased levels of the reactive glycolytic byproduct, methylglyoxal (MGO), and its resulting post-translational modifications (PTMs) compared with nondiabetic patients. To combat this, cells are equipped with the glyoxalase cycle, consisting of two enzymes, glyoxalase 1 (GLO1) and GLO2, to reduce the levels of MGO. Previous work has identified a putative role for MGO in the pathologies associated with obesity. We thus sought to interrogate the role of GLO1 in the context of adipogenesis using GLO1 knockout (GLO1^−/−^) 3T3-L1 preadipocytes. These cells have elevated, physiologically relevant, levels of MGO and MGO-derived PTMs. When differentiated to mature adipocytes, GLO1^−/−^ cells fail to accumulate lipid, despite significant elevations in MGO. We also show a restoration of MGO-derived PTMs in GLO1^−/−^ cells following differentiation. Proteomic analysis reveals significant enrichment in glycolytic and tricarboxylic acid cycle enzymes in WT cells compared with GLO1^−/−^ cells after differentiation. Last, immunoblotting shows decreased AKT phosphorylation and reduced glucose uptake in differentiated GLO1^−/−^ cells. Taken together, our data identify a putative proadipogenic role for GLO1 and MGO in adipogenesis.

Type 2 diabetes (T2D) is a chronic metabolic disease marked by elevated systemic blood glucose leading to insulin resistance (1). Although T2D is a multifaceted disease, obesity is the number one risk factor for the development of insulin resistance and resulting T2D (2). Obesity arises from an expansion in adipose tissue, which can occur in a controlled, or “healthy,” manner, or in a pathogenic, unregulated manner (3). This unhealthy expansion is characterized by an increase in inflammatory markers, reduced metabolic capacity, and altered insulin signaling (3). Adipose expansion occurs through either an increase in total adipocyte number (hyperplasia) or increased adipocyte size (hypertrophy) (4, 5). Given the pervasive nature of obesity and T2D, understanding the mechanisms that dictate healthy and unhealthy adipose tissue expansion is critical (6, 7, 8).

Glycated hemoglobin (HbA1c), or the nonenzymatic addition of glucose to hemoglobin, is an established biomarker for long-term glycemic control (9). In addition to glucose itself, glycation can arise from a myriad of saccharides, including the glycolytic byproduct, methylglyoxal (MGO). MGO and resulting post-translational modifications (PTMs) are significantly elevated in patients with T2D (10). To keep MGO concentrations below the toxic threshold, cells are equipped with the glyoxalase cycle, consisting of two enzymes, glyoxalase 1 (GLO1) and GLO2 (Fig. 1*A*) (11, 12). As diabetics have disruptions in their ability to metabolize glucose, elevated levels of MGO, and the resulting glycation products, have been reported (13, 14). MGO is thought to be generated through spontaneous degradation of the triose phosphate, dihydroxyacetone phosphate (DHAP) (15). MGO is a highly reactive dicarbonyl that can react with Arg and Lys residues on proteins to form MGO-derived PTMs (16). These PTMs are believed to be toxic, altering protein structure and initiating stress signaling pathways (17, 18, 19, 20). Yet, despite these associations, the exact mechanism that these PTMs play in protein function remains unknown. At the center of this is a reliance on studies that require a bolus administration of supraphysiological concentrations of MGO. For example, bolus administration of MGO has been shown to drive adipocyte proliferation in an AKT1 phosphorylation–dependent mechanism (21). Furthermore, MGO-derived PTMs disrupt adipose tissue integrity, ultimately compromising insulin responsiveness, following high levels of dietary MGO supplementation in rats (22, 23). Thus, it is difficult to assess the (patho)physiological role of MGO with a strong reliance on bolus dosing of MGO.

**Figure 1:**
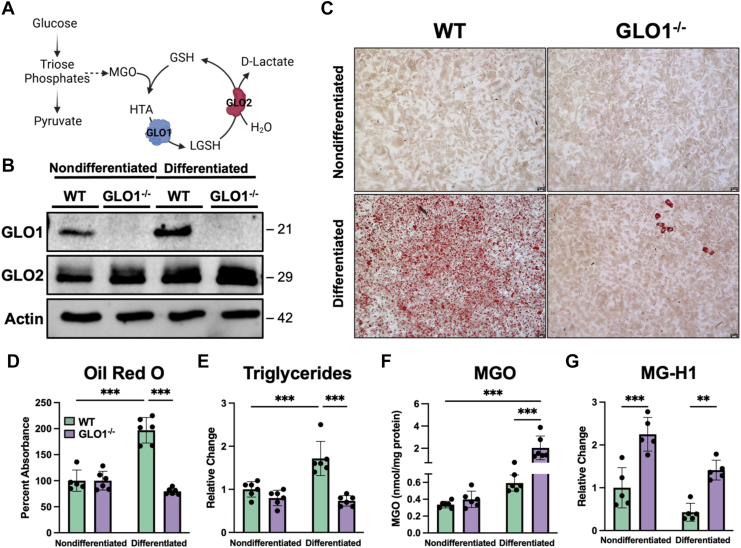
**3T3-L1 GLO1^−/−^ cells do not accumulate lipid droplets following differentiation.***A* and *B*, the glyoxalase cycle and GLO1^−/−^ cells used for differentiation. *C*, Oil Red O, a well-established differentiation marker, staining on WT and GLO1^−/−^ cells with and without differentiation shows an accumulation of lipid droplets in WT cells after differentiation, which is absent in GLO1^−/−^ cells. *D*, quantification of Oil Red O confirms the accumulation of lipid droplets in WT cells following differentiation but not in GLO1^−/−^ cells. *E*, while WT cells accumulate triglycerides appropriately, GLO1^−/−^ triglycerides are reduced. *F*, MGO levels are elevated in GLO1^−/−^ cells after differentiation but not in WT cells. *G*, quantification of the MGO-derived PTM, MG-H1, shows an elevation in GLO1^−/−^ cells. These PTMs are decreased in WT cells following differentiation and are elevated in GLO1^−/−^ after differentiation. N = 5 to 6, ± SD. ∗∗*p* < 0.01, ∗∗∗*p* < 0.001 by two-way ANOVA. GLO1/2, glyoxalase 1/2; HTA, hemithioacetal; (L)GSH, (lactoyl)glutathione; MG-H1, methylglyoxal-hydroimidazolone 1; MGO, methylglyoxal; PTM, post-translational modification.

Herein, we demonstrate that GLO1^−/−^ 3T3-L1 cells fail to differentiate into mature adipocytes. We show that in the absence of GLO1, MGO and MGO-derived PTMs are elevated when compared with WT counterparts. Ablation of GLO1 ultimately rewires cellular metabolism, leading to a significant decrease in glycerol-3-phosphate, pointing to a reduced capacity for *de novo* triglyceride synthesis. Quantification of nutrient levels reveals increases in the ATP:AMP + ADP ratio in GLO1^−/−^ cells, demonstrating an increase in energy catabolism. Moreover, we show disrupted AKT phosphorylation in cells lacking GLO1. Last, expression of essential transcription factors for adipocyte differentiation is absent in GLO1^−/−^ cells. Taken together, we define a novel, *proadipogenic* role for GLO1 in adipogenesis, highlighting the potential importance of MGO and MGO-derived PTMs in homeostasis.

## Results

### Characterization and generation of 3T3-L1 GLO1^−/−^ cells

To circumvent a reliance on bolus dosing studies to investigate the role of MGO in adipogenesis, we generated GLO1^−/−^ 3T3-L1 fibroblasts (Fig. S1*A*). These cells display an inability to metabolize exogenous MGO, as observed through elevations in MGO and MGO-derived PTMs (carboxyethyllysine; methylglyoxal-hydroimidazolone 1 [MG-H1]; and carboxyethylarginine) (Fig. S1, *B*–*E*). These data are consistent with the reported actions of GLO1 and thus validate this as a model to study the endogenous production of MGO in a physiologically relevant context (24, 25).

### GLO1^−/−^ 3T3-L1 cells fail to differentiate into mature adipocytes

We next sought to investigate the role of GLO1, and by proxy MGO, in a cell culture model for adipogenesis (Fig. 1, *A* and *B*). Using established methods for the differentiation of 3T3-L1 preadipocytes, we induced differentiation in WT and GLO1^−/−^ cells (Fig. 1*C*) (26). As shown in Figures 1, *C*–*E* and S2, GLO1^−/−^ cells display a marked reduction in lipid and triglyceride accumulation. Because we demonstrated the same phenotype among clones, we chose GLO1^−/−^ #6 for all future experiments in this article. Quantification of MGO (Fig. 1*F*) and MGO-derived PTMs (Figs. 1*G* and S3) reveals a significant increase in MGO, MG-H1, and carboxyethylarginine in differentiated GLO1^−/−^ cells when compared with WT counterparts. We demonstrate no significant elevations in MGO levels in nondifferentiated cells, regardless of genotype (Fig. 1*F*). This is consistent with our previous reports (24, 25), where no changes in MGO are observed in the absence of external stress/stimuli. Here, the stress represents differentiation to adipocytes. This lack of elevation may also explain the increase in the MGO-derived PTM, MG-H1 in GLO1^−/−^ nondifferentiated cells (Fig. 1*G*). Collectively, these data contradict previous correlative reports demonstrating increased MGO and MGO-derived PTMs in patients with diabetes and obesity (27). In contrast, our results reveal a significant reduction in adipocyte differentiation whilst maintaining increased levels of MGO and MGO-derived PTMs in the absence of GLO1. This phenotype led us to hypothesize that GLO1 may be a critical *proadipogenic* enzyme dictating adipocyte differentiation through altered cellular metabolism and the maintenance of MGO-derived PTMs on key metabolic enzymes.

### Metabolic enzymes are enriched in mature WT adipocytes compared with GLO1^−/−^ cells

**Figure 2:**
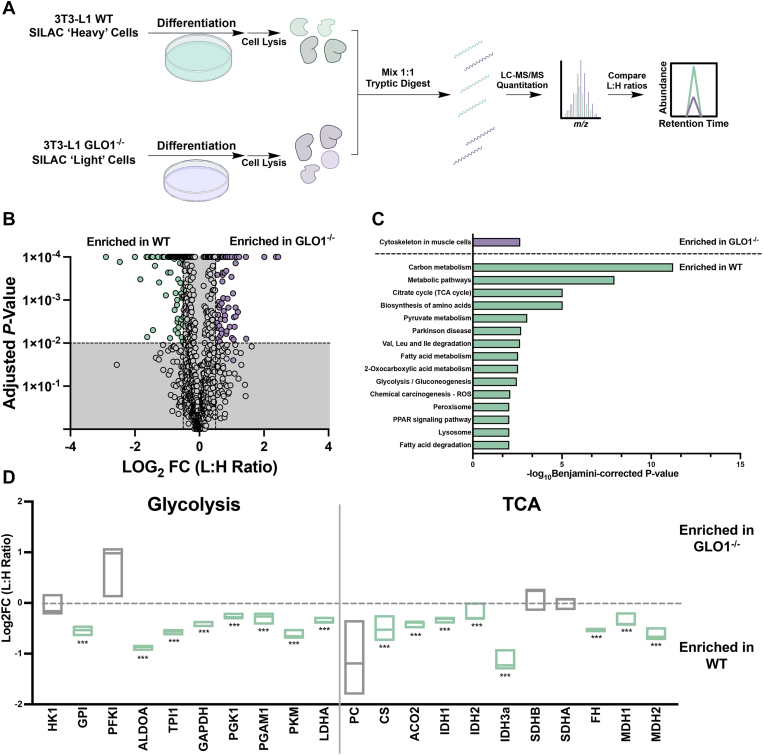
**Metabolic enzymes are reduced in GLO1^−/−^ cells following differentiation.***A*, an overview of the method. *B*. Volcano plot for the proteins identified in each cell line. Proteins shown display a Log2FC >±0.5 and a Benjamini–Hochberg–corrected *t* test (*p* value) <0.01. *C*, KEGG analysis was performed on the proteins enriched in WT and GLO1^−/−^ cells following differentiation, revealing metabolic pathways that are enriched in both genotypes. Pathways shown have a Benjamini-corrected *p* value <0.01. *D*, mapping of the glycolytic and TCA cycle enzymes that were identified, where *green* is significant enrichment in WT differentiated and *gray* signifies no significant enrichment. N = 3, ±SD. ∗∗∗*p* < 0.001 by one-way ANOVA. FC, fold change; GLO1, glyoxalase 1; KEGG, Kyoto Encyclopedia of Genes and Genomes; TCA, tricarboxylic acid.

We next employed a proteomics approach to quantify changes in protein expression resulting from GLO1 ablation in differentiated cells (Fig. 2*A*). Using a stable isotope labeling of amino acids in cell culture (SILAC) approach, we identified 171 proteins in three replicates (Fig. 2*B*, Tables S1, S2 and S4). A Kyoto Encyclopedia of Genes and Genomes analysis of these enriched proteins reveals a marked elevation in pathways involved with primary metabolism (glycolysis, tricarboxylic acid cycle, carbon metabolism, amino acid metabolism, etc.) as well as fatty acid metabolism in differentiated WT cells, which were absent in GLO1^−/−^ cells (Fig. 2*C* and Tables S3 and S5).

Further investigation demonstrates a significant decrease in glycolytic and tricarboxylic acid cycle enzymes in differentiated GLO1^−/−^ cells (Fig. 2*D*). Interestingly, with enzymes involved in fatty acid metabolism (ATP citrate lyase and fatty acid synthase), no significant difference in enrichment between WT and GLO1^−/−^ cells was observed (Fig. S4*A*). We also demonstrate an enrichment of glycerol-3-phosphate dehydrogenase 1 (GPD1), acetyl-CoA carboxylase (ACC), and AMP-activated kinase (AMPK) in WT cells but not GLO1^−/−^ cells. Last, mapping of lipid-binding proteins reveals a significant enrichment in fatty acid–binding proteins in WT cells, which are markers for mature adipocytes (Fig. S4*B*).

### Glycerol-3-phosphate and acetyl-CoA are decreased in GLO1^−/−^ cells following differentiation

**Figure 3:**
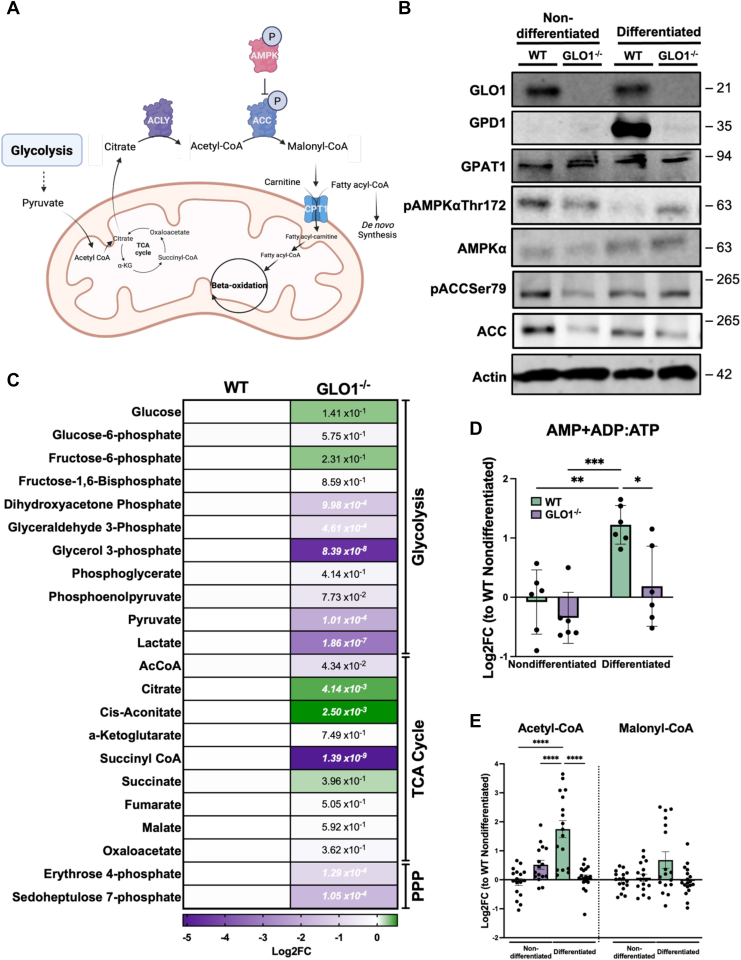
**Differentiated GLO1^−/−^ cells have reduced glycerol-3-phosphate and fatty acid synthesis intermediates.***A*, schematic for the synthesis and oxidation of fatty acids. *B*, GPD1 expression is reduced in GLO1^−/−^ cells compared with WT. AMPK activation is not significantly different between genotypes. ACC phosphorylation is increased in differentiated WT cells. Representative blot. N = 6. *C*, GLO1^−/−^ cells have reduced triose phosphates and G-3-P compared with WT counterparts following differentiation. N = 6. Values are reported as Log2FC, normalized to WT differentiated. Values shown are from a Welch’s *t* test comparing WT to GLO1^−/−^ cells in the differentiated state. *D*, AMP + ADP:ATP ratios of WT and GLO1^−/−^ cells after differentiation demonstrate higher levels of ATP in GLO1^−/−^ cells compared with WT cells and similar to nondifferentiated cells. N = 6, ±SD. ∗*p* < 0.05, ∗∗*p* < 0.01, and ∗∗∗*p* < 0.001 by two-way ANOVA. Values are reported as Log2FC, normalized to WT nondifferentiated. *E*, quantification of acetyl-CoA and malonyl-CoA reveals an increase in acetyl-CoA in WT cells following differentiation, which is not present in GLO1^−/−^ cells. N = 17 to 18, ±SEM. ∗∗∗*p* < 0.001, ∗∗∗∗*p* < 0.0001 by two-way ANOVA. Values are reported as Log2FC, normalized to WT nondifferentiated. ACC, acetyl-CoA carboxylase; AMPK, AMP-activated kinase; FC, fold change; G-3-P, glycerol-3-phosphate; GLO1, glyoxalase 1; GPD1, glycerol-3-phosphate dehydrogenase 1.

Adipocyte differentiation is dependent on glucose (4) and fatty acid synthesis (Fig. 3*A*). Thus, we first evaluated the expression of key metabolic enzymes involved in triglyceride biosynthesis (Fig. 3*B*). GLO1^−/−^ cells have reduced expression of the rate-limiting enzyme in triglyceride biosynthesis, GPD1. This enzyme converts the glycolytic intermediate (and proposed MGO precursor), DHAP, to glycerol-3-phosphate (G-3-P), thus linking glycolysis with *de novo* triglyceride synthesis. To determine if the effects on GPD1 expression result from reduced transcription, we performed quantitative RT–PCR (qRT–PCR) to quantify GPD1 mRNA and observe a near complete ablation of transcript in GLO1^−/−^ cells (Fig. S5*A*). In addition to glycerol, triglycerides also require free fatty acids for their formation (28). This is regulated by AMPK and ACC. AMPK is critical for maintaining energy homeostasis and is a master regulator of cell signaling, including adipocyte differentiation (29). We thus performed immunoblotting for proteins involved in this pathway, revealing no significant differences in the phosphorylation of AMPKα at Thr172 in GLO1^−/−^ following differentiation compared with WT cells (Figs. 3*B* and S5, *B* and *C*). Quantification of ACC phosphorylation at Ser79 reveals a similar trend, where there are no differences between differentiated WT and GLO1^−/−^ cells (Figs. 3*B* and S5*C*). We next quantified primary metabolites in WT and GLO1^−/−^ cells after differentiation (Figs. 3*C*, S6 and S7). As shown in Figure 3*C*, the triose phosphates, DHAP and glyceraldehyde 3-phosphate, are significantly reduced in GLO1^−/−^ cells. Most notably, we show a significant decrease in G-3-P in GLO1^−/−^ cells, a metabolite necessary for triglyceride synthesis and the product of GPD1 activity, which is lost in GLO1^−/−^ cells. This is further corroborated with the data presented in Figure 1*E*, where a significant reduction in triglycerides is observed. We next quantified AMP, ADP, and ATP as these metabolites regulate AMPK activation and thus fatty acid synthesis (Fig. 3*D*). We demonstrate an increase in this ratio in WT cells and restored balance in GLO1^−/−^ cells, even though we do not observe an increase in pAMPK (Figs. 3*B* and S5*B*). Last, as the functional readout for ACC activity, we measured CoAs and carnitines, revealing increased acetyl-CoA levels in WT cells following differentiation that is not present in GLO1^−/−^ cells (Figs. 3*E* and S8). Although we observe no significant differences in immunoblotting in neither pAMPK nor pACC, our metabolomic quantification clearly demonstrates alterations in this important pathway. Interestingly, we observe no significant differences in malonyl-CoA levels between genotypes. Overall, we observe a reduction in numerous CoA and carnitine species in GLO1^−/−^ cells following differentiation compared with WT counterparts (Fig. S8).

### AKT signaling is disrupted in GLO1^−/−^ cells following differentiation

**Figure 4:**
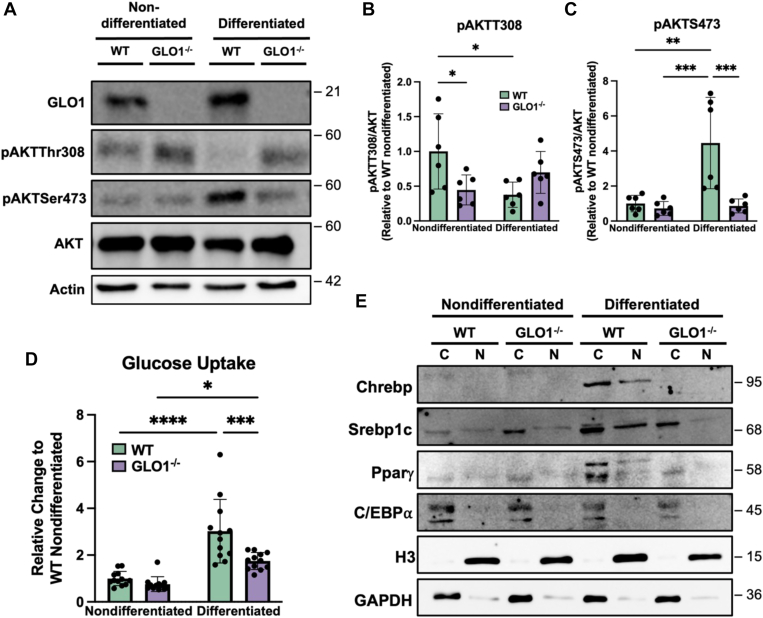
**AKT signaling is altered in GLO1^−/−^ cells following differentiation.***A*–*C*, representative immunoblotting (*A*) and quantification (*B* and *C*) reveals marked decreases and increases at active phosphorylation sites on AKT in GLO1^−/−^ cells following differentiation. *D*, glucose uptake is reduced in differentiated GLO1^−/−^ cells compared with WT. N = 6 (*B* and *C*), N = 12 (*D*) ∗*p* < 0.05, ∗∗*p* < 0.01, ∗∗∗*p* < 0.001, ∗∗∗∗*p* < 0.0001 by two-way ANOVA. *E*, the transcription factors, ChREBP and PPARγ, are only expressed in WT differentiated cells and not in GLO1^−/−^ cells. Probing of Srebp1c displays no translocation into the nucleus in GLO1^−/−^ cells after differentiation, compared with WT cells. ChREBP, carbohydrate response element binding protein; GLO1, glyoxalase 1; PPARγ, peroxisome proliferator–activated receptor gamma.

In addition to AMPK, mammalian target of rapamycin (mTOR) functions as a critical nutrient signaling enzyme (30). Similar to pAMPKα, we show no significant differences in the phosphorylation of mTOR (Fig. S9). As AMPK regulates mTOR (29), we predicted decreases in phosphorylation in GLO1^−/−^ cells because of the AMP + ADP/ATP ratios; however, this was not demonstrated. Importantly, we show decreased phosphorylation of AKT on Ser473 and an increase on Thr308 in GLO1^−/−^ cells (Fig. 4, *A*–*C*). AKT activation is necessary for adipogenesis and glucose–insulin signaling (31). pAKTS473 is also considered a target of mTORC2 (32). Although mTOR phosphorylation shows no significant differences, pAKTSer473 clearly demonstrates a reduction in GLO1^−/−^ cells. In contrast, pAKTThr308 is increased in GLO1^−/−^ cells compared with WT. Due to the role of AKT in glucose signaling, we performed a glucose uptake assay and, we demonstrate a significant reduction in glucose uptake in GLO1^−/−^ cells when compared with WT counterparts (Fig. 4*D*).

Last, we probed for transcription factors necessary for adipocyte differentiation and maturation (Fig. 4*E*). Using subcellular fractionation, we show induction of neither peroxisome proliferator–activated receptor gamma (PPARγ) nor carbohydrate response element (carbohydrate response element binding protein [ChREBP]) in GLO1^−/−^ cells following differentiation. PPARγ is regulated through insulin signaling and is necessary for adipocyte differentiation (33). ChREBP is glucose responsive and required for lipid and glucose metabolism (34). Furthermore, as the primary regulator of *de novo* sterol biosynthesis, Srebp1c (sterol response binding element) remains sequestered in the cytosol, with no detectable expression in the nucleus in the GLO1^−/−^cells (35). Altogether, our data indicate that GLO1 ablation disrupts insulin signaling, ultimately reducing glucose uptake and metabolism and inhibiting adipogenesis in 3T3-L1 cells.

## Discussion

In this article, we describe GLO1 as a novel proadipogenic gene. There are many facets that govern alterations in cell metabolism, and thus, further investigation must take place to increase understanding of the molecular mechanisms that govern disease progression. MGO metabolism has been heavily implicated in the progression of T2D, and studies have mainly focused on correlations between increased MGO and MGO-derived PTMs and disease. To date, many whole-body GLO1 knockout animals have been developed, including *Drosophila*, *Caenorhabditis elegans*, zebrafish, and mice (36, 37, 38, 39, 40). In mice, GLO1 knockout has no profound phenotypic effect at the metabolic level, where compensatory detoxification mechanisms coped with the levels of MGO and MGO-derived PTMs (39). Furthermore, many of these *in vivo* studies rely on bolus administration of MGO, not exclusively on GLO1 (41). In contrast, however, knockdown and inhibition does mimic a diabetic phenotype in mice (42, 43). However, as alluded to, these studies provide little insight into a causative mechanism of MGO-induced insulin resistance. It is for this reason that we sought to investigate the regulatory role that GLO1 plays in adipogenesis.

MGO has been shown to promote adipogenesis through phosphorylation of AKT1 in 3T3-L1 cells (21). Thus, we initially hypothesized that ablation of GLO1, and thus increases of MGO, would promote adipogenesis in 3T3-L1 cells. Figure 1, however, reveals a failure of these cells to mature to adipocytes in the absence of GLO1. This is confirmed *via* Oil Red O staining and triglyceride quantification (Fig. 1). To further characterize the role of GLO1 in adipogenesis, we quantified the levels of MGO and MGO-derived PTMs, given their implications in obesity. We observe that, despite a failure to undergo adipogenesis, GLO1^−/−^ cells have a significant elevation in MGO and MGO-derived PTMs (Fig. 1).

Quantitative metabolomics further explains the observed phenotype in GLO1^−/−^ cells following differentiation. We show a significant decrease in G-3-P, a critical metabolite in triglyceride synthesis, in GLO1^−/−^ cells following differentiation (Fig. 3*C*). This decrease in G-3-P explains the lack of lipid droplets observed in GLO1^−/−^ cells. Furthermore, we observed a decrease in GPD1 expression both at the protein and mRNA levels (Figs. 3*B* and S5*A*), with no alterations in other enzymes in the triglyceride synthesis pathway, demonstrating a specificity for regulation of GPD1 by GLO1 in the context of adipocyte differentiation. Our data suggest that GPD1 expression is dependent on the expression of GLO1. Alterations in GPD1 are implicated in many metabolic disorders, and this enzyme is critical for cell health (44). Indeed, GLO1 and GPD1 have been shown to regulate conditions of high MGO in yeast (45). To our knowledge, this is the first demonstration of GLO1 regulating GPD1 expression in the context of adipocyte differentiation.

AMPK regulates numerous facets of cell metabolism, cell growth, and differentiation (29). Perhaps the most well-defined function is to inhibit triglyceride and fatty acid synthesis, thus promoting beta oxidation (6). In addition, AMPK activation has been shown to induce glucose uptake in 3T3-L1 adipocytes (46). We thus interrogated the contribution of AMPK in the context of GLO1, revealing no significant alterations despite higher concentrations of AMP and ADP levels in WT cells compared with GLO1^−/−^ cells (Fig. 3*D*). In this context, WT differentiated cells have increased levels of AMP, which should activate AMPK and ultimately increase fatty acid oxidation for energy usage. When ATP levels are high (GLO1^−/−^ cells), AMPK activation is not needed, thus reducing fatty acid oxidation. Fatty acid oxidation is also controlled through carnitine palmitoyl transferase 1. Malonyl-CoA inhibits the transport of fatty acids into the mitochondria *via* binding to the active site channel of carnitine palmitoyl transferase 1, thus reducing the capacity for fatty acid oxidation. Interestingly, we did not observe any significant differences in malonyl-CoA levels between genotypes (Fig. 3*E*). We did, however, observe a significant increase in acetyl-CoA levels in WT cells following differentiation, which was not present in GLO1^−/−^ cells (Fig. 3*E*). This is also corroborated through the increase in ACC phosphorylation.

Previous studies have highlighted the importance of AKT phosphorylation in the differentiation of adipocytes. This is particularly relevant in the context of MGO where a bolus administration was found to promote adipogenesis in 3T3-L1 cells (21). Conversely, ablation of GLO1 results in a reduction in AKTSer473 phosphorylation after differentiation (Fig. 4*C*). This site increases the enzyme activity of AKT, further inducing its downstream effects (47). In contrast, we show an increase in phosphorylation of Thr308 on AKT in GLO1^−/−^ cells (Fig. 4, *A* and *B*). AKT phosphorylation at Thr308 is induced by insulin signaling, and its activity is necessary for GLUT4 translocation to the membrane for glucose uptake (48). Figure 4*D* reveals a significant reduction in glucose uptake in GLO1^−/−^ cells, despite AKTThr308 phosphorylation. In contrast, our data suggest that GLO1 ablation prevents phosphorylation of AKTSer473.

**Figure 5:**
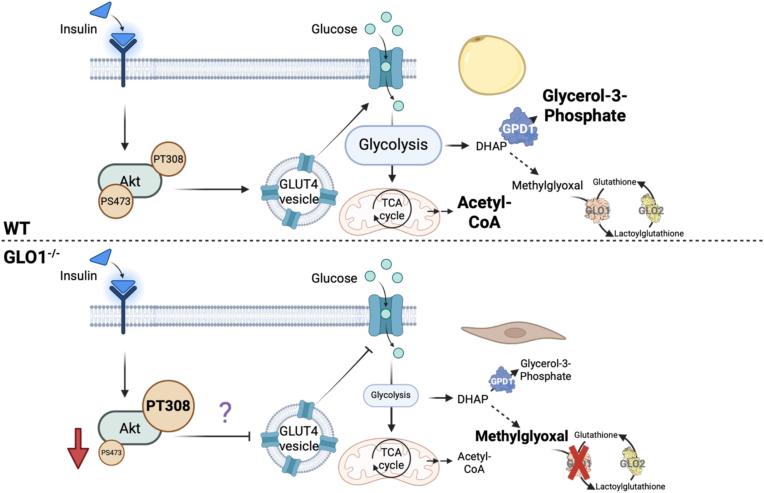
**Phenotypic alterations following GLO1 ablation in 3T3-L1 cells.** Under normal circumstances, insulin signaling leads and AKT phosphorylation, GLUT4 translocation, and glucose uptake. In contrast, when GLO1 is not present, AKT phosphorylation is altered, and glucose uptake is decreased. This leads to altered glucose metabolism and no accumulation of lipid droplets. GLO1, glyoxalase 1.

Taken together, we demonstrate a putative role for GLO1 in the regulation of adipogenesis. We hypothesize that GLO1 ablation prevents AKT phosphorylation at Ser473 through a yet-to-be defined mechanism, decreasing glucose uptake and thus differentiation (Fig. 5). In this article, we provide numerous insights into how GLO1 ablation affects 3T3-L1 preadipocyte differentiation at the proteomic, metabolomic, and transcriptomic levels. Although phenotypically it appears that GLO1 ablation may protect cells from lipid accumulation, additional studies are required to elucidate the full mechanism.

## Experimental procedures

### Cell culture

NIH/3T3-L1 cells were purchased from American Type Culture Collection and cultured in high-glucose Dulbecco's modified Eagle's medium (DMEM) supplemented with 10% calf bovine serum. Cells were incubated at 37 °C under 5% CO_2_.

### CRISPR–Cas9

Guide RNA oligonucleotides were designed to target restriction enzyme recognition sites in exon 1 of the *GLO1* locus and ligated into the pSpCas9(BB)-2A-GFP plasmid according to Cong *et al.* (22). TGAAAGCGGTCTCATCAGTG was inserted into the plasmid to target GLO1. To generate G*LO1*^*−/−*^, 2 × 10^5^ NIH/3T3-L1 cells were plated in 2 ml DMEM supplemented with 10% calf bovine serum in 6-well plates. The following day, 5 μg of each construct was combined with 10 μl Lipofectamine 2000 (Life Technologies) reagent in 1 ml Opti-MEM and incubated at room temperature for 15 min. The DMEM was replaced with the plasmid–Lipofectamine solution, and the cells were incubated at 37 °C for 24 h. The medium was then replaced, and cells were allowed to recover for 24 h at 37 °C. Cells were then harvested for single cell sorting GFP-positive cells. Colonies were verified for GLO1 knockout *via* immunoblotting.

### Differentiation of 3T3-L1 preadipocytes into mature adipocytes

Differentiation was executed as previously described (26). Cells were seeded in respective plates (6-well for MGO and PTM quantification, immunoblotting, proteomics, qRT–PCR, and metabolomics, Oil Red O staining; 96-well for glucose uptake) and cultured as above until 100% confluence. Two days postconfluence, differentiation was initiated by replacing the culture media with methylisobutylxanthine, dexamethasone, insulin induction media containing methylisobutylxanthine (0.5 mM final in dimethyl sulfoxide), dexamethasone (1 μM final in dimethyl sulfoxide), and insulin (10 μg/ml final) (day 0). On day 3, media were removed and replaced with media containing only insulin (10 μg/ml final). Media were replaced every 3 days until day 12. Control cells (nondifferentiated) were seeded in the same manner, but no induction media were added. All experiments were performed on day 12 unless otherwise specified.

### Quantification of intracellular MGO

3T3-L1 WT and GLO1^−/−^ cells were seeded at 4 × 10^5^ cells in 6-well plates overnight. The following day, media were removed, and the cells were treated with vehicle or 250 μM MGO for 6 h. After treatment, media were removed, and cells were washed 1X with ice-cold PBS and scraped into 1 ml of ice-cold PBS. After centrifugation (1000*g*, 5 min), PBS was removed, and cell pellets were resuspended in ice-cold 80:20 MeOH:H_2_O (250 μl) containing 2.5 nmol ^13^C-MGO and briefly sonicated into solution. Protein was then precipitated >1 h at −80 °C. Samples were then centrifuged (14,000*g*, 10 min) and 10 mM *O-*phenylenediamine was added (10 μl), and samples were derivatized as previously described, rotating in the dark for 1.5 h (25). After derivatization, samples were centrifuged to remove any precipitate and plated. Samples were chromatographed (6 μl) using a Shimadzu LC system equipped with a 50 × 2.1 mm, 5 μm particle diameter Atlantis C_18_ column (Waters) at a flow rate of 0.500 ml min^-1^. Buffer A (0.1% formic acid in water) was held at 20% for 0.50 min, and then a linear gradient to 98% solvent B (0.1% formic acid in acetonitrile [ACN]) was applied over the next 3 min. The column was held at 98% B for 2 min and then 20% B for 0.5 min and equilibrated to 80% A for 1.5 min between runs. Multiple reaction monitoring (MRM) was conducted in positive mode using an AB SCIEX 6500 QTRAP with the following transitions: *m/z* 145.0 → 77.0 (analyte); *m/z* 148.0 → 77.0 (^13^C_3_-MGO, internal standard [IS]) (24).

### Quantification of MGO-derived PTMs

3T3-L1 WT and GLO1^−/−^ cells were seeded at 4 × 10^5^ cells in 6-well plates overnight. The following day, media were removed, and the cells were treated with vehicle or 250 μM MGO for 6 h. Following treatment, media were removed, and cells were washed 1X with ice-cold PBS and scraped into 1 ml of ice-cold PBS. After centrifugation (1000*g*, 5 min), PBS was removed, and cell pellets were resuspended in lysis buffer containing 100 mM Tris–HCl, 150 mM NaCl, 10 mM NaF, 1% Triton X-100, pH = 7.4. Protein concentration was determined *via* bicinchoninic acid (BCA), and 100 μg of protein was precipitated in ice-cold acetone (500 μl) overnight at −20 °C. Protein was pelleted (14,000*g*, 10 min), and pellets were allowed to air dry. Ammonium bicarbonate (50 mM; 65 μl) was added to each tube. An IS mixture (10 μl; below) and 10 μl of sequencing-grade trypsin (0.1 mg/ml) was added to each sample. Samples were then incubated at 37 °C for 3 h. Following incubation, samples were boiled (95 °C, 10 min), cooled, spiked with 1.5 μg aminopeptidase (in 10 μl), and incubated overnight at 37 °C. The following day, samples were again boiled (95 °C, 10 min), cooled, and spiked with 1:1 H_2_O:heptafluorobutryic acid (HFBA, 7.66 M). Samples were chromatographed (12 μl) using an AB SCIEX 6500 QTRAP LC at a flow rate of 0.450 ml min^-1^ with a 2.1 × 150 mm, 3.5 μM Eclipse XD8-C_8_ column (Agilent). Buffer A: 10 mM HFBA in water, buffer B: 10 mM HFBA ACN. The following gradient was applied: 1 min, 2.5% B; 8 min, 50% B; 8.5 min, 98% B; 12 min, 98% B; and 12.5 min, 2.5% B. The column was equilibrated at 2.5% B for 3 min between runs. MRM was conducted in positive mode using the parameters below (Table 1). Analytes were quantified against their ISs and normalized to Leu.

**Table 1:** MS parameters for PTM analysis

Q1	Q3	Analyte	CE
247	70	CEA	47
248	70	^13^C-CEA	47
219	84	CEL	27
223	88	CEL-d_4_	27
229	70	MG-H1	42
230	70	^13^C-MG-H1	42
132	86	Leu	14
138	91	^13^C_6_^15^N Leu	14

CE, collision energy; CEA, carboxyethylarginine; CEL, carboxyethyllysine; MG-H1, methylglyoxal-hydroimidazolone; Q1, parent ion; Q3, fragment ion.

### Quantification of intracellular MGO and MGO-derived PTMs in differentiated cells

On day 12 of with/without differentiation, cells were harvested as described above.

### Oil Red O staining and quantification

On day 12 of with/without differentiation, the cells were stained as described below. Oil Red O was prepared as follows: Oil Red O was dissolved in 100% isopropanol at 3 mg/ml and allowed to sit for 20 min. The working solution was then made at 3:2 Oil Red O:H_2_O and filtered using a 0.2 μM syringe filter. Solution was prepared 15 min before use. For staining, media were removed, and cells were washed 2X with cold PBS. Formalin (10%) was then added to cells and allowed to fix for 30 to 60 min. After fixing, cells were washed 2X with ddH_2_O, and 60% isopropanol (in H_2_O) was added for 5 min. After incubation, 60% isopropanol was then removed, and the filtered Oil Red O solution was added and allowed to stain for 20 min. Solution was then removed, and cells were washed continuously with ddH_2_O until stain was removed. Hematoxylin was then added for 1 min and rinsed until the stain was completely removed. Water was then added to cells and imaged. After imaging, stain was extracted with 100% isopropanol and measured at 490 nm.

### Triglyceride quantification

Cells were plated and differentiated as described above. On day 12, media were removed, and cells were washed with 1 ml ice-cold PBS. Cells were then scraped in 1 ml PBS, pelleted (1000*g*, 5 min, 4 °C), and resuspended in 450 μl PBS. Samples were sonicated into solution, and 2.25 ml of 2:1 chloroform:MeOH was added. After vigorous vortexing, H_2_O (1 ml) was added. Samples were centrifuged (2200*g*, 5 min, room temperature), and the top aqueous layer was transferred and dried under nitrogen. Samples were resuspended in 95% EtOH (200 μl) and sonicated into solution. Triglycerides were quantified according to the manufacturer’s protocol (Sekisui Diagnostics).

### SDS-PAGE and immunoblotting

Cells were plated, differentiated, and harvested as described above. Samples were then prepared and denatured in SDS loading buffer and boiled at 95 °C for 5 min. Proteins were then resolved *via* SDS-PAGE and transferred to nitrocellulose membranes and blocked in Intercept Blocking Buffer for >30 min at room temperature until probing overnight with primary antibody at 4 °C as described: GLO1 (1:1000 dilution; Abcam, Ab81461); GLO2 (1:1000 dilution; Invitrogen, A5-80682); GPD1 (1:1000 dilution; Abcam, Ab153902); GPAT1 (1:1000 dilution; Millipore, ABS764); pAMPKαThr172 (1:1000 dilution; Cell Signaling Technologies, 2535T); AMPKα (1:1000 dilution; Cell Signaling Technologies, 5831T), pACCSer79 (1:1000 dilution; Cell Signaling Technologies, 11818T); ACC (1:1000 dilution; Cell Signaling Technologies, 3676T); pmTORS2481 (1:1000 dilution; Cell Signaling Technologies, 2974T); pmTORS2884 (1:1000 dilution; Cell Signaling Technologies, 5536T); mTOR (1:1000 dilution; Cell Signaling Technologies, 2983T); pAKT473 (1:1000 dilution; Cell Signaling Technologies, 4060T); pAKTT308 (1:1000 dilution; Cell Signaling Technologies, 13038T); AKT (1:1000 dilution; Cell Signaling Technologies, 9272S); Actin (1:10,000 dilution; Sigma, A1978); ChREBP (1:1000 dilution; Cell Signaling Technologies, 58069S); Srebp1c (1:1000 dilution; ProteinTech, 14088); PPARγ (1:500 dilution; Cell Signaling Technologies, 2430S), C/EBPα (1:1000 dilution; Cell Signaling Technologies, 2295S); H3 (1:5000 dilution; Cell Signaling Technologies, 4499T); and GAPDH (1:1000 dilution; Cell Signaling Technologies, 5174T). The following day, membranes were rinsed 3X with Tris-buffered saline with Tween-20 and incubated with infrared secondary antibody (Li-COR; 1:5000 dilution) for 45 min at room temperature. After rinsing 3X with Tris-buffered saline with Tween-20, membranes were imaged using c600 Azure Imaging System. Images were quantified using ImageJ (National Institutes of Health).

### Targeted metabolomics in differentiated 3T3-L1 cells

Cells were plated and differentiated as described above. On day 12, cells were gently washed 2X with warm PBS. Ice-cold 80:20 MeOH:H_2_O (1 ml) containing 1 nmol glucose-6-phosphate IS and 0.25 nmol Lys IS was added, and metabolites were extracted for 15 min at −80 °C. Cells were then scraped and washed 1X with ice-cold 80:20 MeOH:H_2_O. Samples were pelleted (4000*g*, 5 min, 0 °C), and supernatant was transferred to new tubes. Samples were dried under nitrogen and resuspended in 80:20 MeOH:H_2_O (120 μl). Metabolites were chromatographed (6 μl) using a Shimadzu LC system equipped with a 2.1 mm × 100 mm, 3.5 μm particle diameter XBridge Amide column (Waters) at a flow rate of 0.50 ml min^-1^. Buffer A (15 mM ammonium bicarbonate in ddH_2_O, pH = 9.0) and buffer B (15 mM ammonium bicarbonate in 90% ACN, pH = 9.0) were chromatographed as follows: 1.0 min, 2% A, B curve of 2; 8.0 min, 90% A; 9.5 min, 90% A; 10.0 min, 2% A; 10.5 min, 2% A. The column was equilibrated at 98% B for 2 min between runs. MRM was performed in positive and negative ion mode using an AB SCIEX 6500 QTRAP with the parameters defined in Table 2. Negative compounds were quantified against glucose-6-phosphate IS. Positive values were normalized against lysine IS. Values were then normalized to total protein determined *via* BCA. Data are presented as a Log_2_fold change compared with WT differentiated (Figs. 3*C* and S6) or WT nondifferentiated (Fig.S7).

**Table 2:** MS parameters for metabolomics

Q1	Q3	Analyte	CE
179	89	Glucose	−10
259	97	Glucose-6-phosphate	−19
265	97	^13^C_6_ Glucose-6-phosphate (IS)	−19
259.001	97.001	Fructose-6-phosphate	−15
339	97	Fructose-1,6-bisphosphate	−38
169	97	DHAP	−12
169.001	97.001	Glyceraldehyde 3-phosphate	−14
171	79	Glycerol 3-phosphate	−15
185	97	3-Phosphoglycerate	−38
167	79	Phosphoenolpyruvate	−15
87	43	Pyruvate	−10
89	43	Lactate	−14
810	303	Acetyl-CoA	42
191	111	Citrate	−15
173	129	*Cis*-aconitate	−11
145	101	a-Ketoglutarate	−10
868	361	Succinyl-CoA	35
117	73	Succinate	−15
115	71	Fumarate	−10
133	115	Malate	−15
131	87	Oxaloacetate	−10
199	97	Erythrose 4-phosphate	−12
289	97	Sedoheptulose 7-phosphate	−20
155	90	^13^C_6_^15^N_2_ lysine (IS)	25

CE, collision energy; Q1, parent ion; Q3, fragment ion.

### CoA and carnitine quantification

Cells were plated and differentiated as described above. Following differentiation, cells were washed and scraped into ice-cold PBS. Following centrifugation (1000*g*, 5 min, 4 °C), pellets were resuspended in 20% 5-sulfosalicylic acid containing 5 nmol ^13^C_3_-malonyl CoA, 2.5 nmol carnitine-d_3_, and 100 pmol palmitoyl carnitine-d_3_. Samples were briefly sonicated and centrifuged (14,000*g*, 10 min, 4 °C). Supernatant was chromatographed (20 μl) using a Shimadzu LC system equipped with a 150 × 3 mm, 3 μm particle diameter C_18_ column (Phenomenex) at a flow rate of 0.400 ml min^-1^. Buffer A: 10 mM HFBA in water, buffer B: 10 mM HFBA in ACN. The following gradient was used: 2 min, 7.5% B; 12 min, 98% B; 16 min, 98% B; and 16.5 min, 2% B. The column was equilibrated for 2.5 min at 7.5% B between samples. MRM was conducted in positive and negative ion mode according to Table 3. Analytes were quantified against each IS and normalized to total protein, determined *via* BCA.

**Table 3:** MS parameters for acyl-CoA quantification

Q1	Q3	Analyte	CE
855	408	^13^C_3_-Malonyl CoA	−51
852	408	Malonyl CoA	−51
768	261	CoA	45
810	303	C2 CoA	45
824	317	C3 CoA	45
838	331	C4 CoA	45
836	329	C4:1 CoA	45
852	345	C5 CoA	45
866	359	C6 CoA	45
165	85	Carnitine-d_3_	27
162	85	Carnitine	27
204	85	C2 Carnitine	25
218	85	C3 Carnitine	25
232	85	C4 Carnitine	25
230	85	C4:1 Carnitine	25
246	85	C5 Carnitine	25
260	85	C6 Carnitine	25
288	85	C8 Carnitine	27
316	85	C10 Carnitine	27
344	85	C12 Carnitine	27
372	85	C14 Carnitine	27
403	85	C16 Carnitine-d_3_	30
400	85	C16 Carnitine	30

CE, collision energy; Q1, parent ion; Q3, fragment ion.

### Stable isotope labeling of amino acids in cell culture

WT cells were cultured in media containing heavy Lys and heavy Arg until proteins contained heavy isotopes (∼2 weeks). GLO1^−/−^ cells were cultured in media containing the natural isotopes of Lys and Arg. Following incorporation, cells were plated for differentiation as described above in their proper media. On the final day of differentiation, cells were washed and scraped into ice-cold PBS and centrifuged (1000*g*, 5 min, 4 °C). Pellets were then lysed in lysis buffer (above), and protein concentration was determined *via* BCA. WT protein (100 μg; heavy) and 100 μg of GLO1^−/−^ protein (light) was mixed 1:1. Protein was precipitated with ice-cold acetone for 1 h at −20 °C. Protein was pelleted (14,000*g*, 10 min, 4 °C) and resuspended in 50 mM ammonium bicarbonate (50 μl) containing 10 mM DTT for 45 min at 60 °C. Twenty-five microliters of 90 mM iodoacetamide were added and incubated for 30 min at room temperature in the dark. Finally, 25 μl of 40 mM DTT was added and incubated for 30 min at room temperature in the dark. Trypsin (1 μg) was added, and samples were incubated at 37 °C overnight.

### Proteomics methods

LC–MS/MS analysis was carried out using a Q Exactive Plus mass spectrometer (Thermo Fisher Scientific) equipped with an Easy Spray nanoESI source. Peptides were eluted from an Acclaim Pepmap 100 trap column (75 micron ID × 2 cm; Thermo Scientific) onto an Acclaim PepMap RSLC analytical column (75 micron ID × 25 cm; Thermo Scientific) using a 3% to 38% gradient of solvent B (90% ACN, 0.1% formic acid) over 35 min, 38% to 74% solvent B over 10 min, 74% to 100% of solvent B over 5 min, then a hold of solvent 100% B for 10 min, and finally a return to 3% solvent B for 10 min. Solvent A consisted of water and 0.1% formic acid. Flow rates were 300 nl/min using a Thermo Scientific EASY-nLC 1200 System (Thermo Scientific). Data-dependent scanning was performed by the Xcalibur, version 4.0.27.19 software (Thermo Scientific) (49), using a survey scan at 70,000 resolution, scanning *m/z* 360 to 1600, automatic gain control target of 1e5, and a maximum injection time of 65 ms, followed by higher-energy collisional dissociation MS/MS at 27 normalized collision energy, of the 10 most intense ions at a resolution of 17,500, an isolation width of 1.5 *m/z*, an automatic gain control of 1e5, and a maximum injection time of 65 ms. Dynamic exclusion was set to place any selected *m/z* on an exclusion list for 20 s after a single MS/MS. Ions of charge state +1, 7, 8, >8, and unassigned were excluded from MS/MS, as were isotopes.

Tandem mass spectra were searched against *Mus musculus* protein database from SwissProt (July 29, 2020; 17263 entries) to which additional common contaminant proteins (*e.g.*, trypsin, keratins) were appended. All MS/MS spectra were searched using Thermo Proteome Discoverer 2.4.0.305 (ThermoFisher Scientific) considering fully tryptic peptides with up to two missed cleavage sites. Variable modifications considered during the search included methionine oxidation (15.995 Da), cysteine carbamidomethylation (57.021 Da), heavy Lys (+8.014), and heavy Arg (+10.008). Proteins were identified at 99% confidence with XCorr score cutoffs as determined by a reversed database search (50). The protein and peptide identification results were also visualized with Scaffold Q + S v 4.8.7 (Proteome Software, Inc), a program that relies on various search engine results (*i.e.*, Sequest, X!Tandem, MASCOT) and which uses Bayesian statistics to reliably identify more spectra (51). Protein identifications were accepted that passed a minimum of two peptides identified at 0.1% peptide false discovery rate and 90% to 99.9% protein confidence by the Protein Profit algorithm within Scaffold. SILAC quantification was performed using Proteome Discoverer (ThermoFisher), and only proteins with reported H:L ratios in three replicates were considered positive identifications. Proteins reported were found in three replicate samples (Figs 2 and S4*B*). In Fig. S4*A*, proteins were reported if found in at least one sample. These were then validated *via* immunoblotting (Fig. 3*B*).

### Glucose uptake

Glucose uptake was measured according to the manufacturer’s protocol (Cayman Chemicals; item no.: 600470). Briefly, cells were differentiated as described above. On the final day of with/without differentiation, cells were glucose starved for 24 h. Following starvation, 2-NBDG (100 μl) was added to cells at a final concentration of 200 μg/ml for 10 min. Plate was centrifuged at 400*g* for 5 min. Supernatant was removed, and cell-based assay buffer was added (200 μl). Plate was centrifuged (400*g*, 5 min), and cell-based assay buffer was added again (100 μl). Plate was immediately measured with a plate reader (excitation/emission: 485 nm/535 nm). Values are normalized to nondifferentiated WT.

### Quantitative RT–PCR

Cells were plated, differentiated, and harvested as described above. RNA was extracted according to the manufacturer’s protocol (Qiagen RNeasy Plus Mini Kit; catalog no.: 74134). Complementary DNA was generated from Verso cDNA Synthesis Kit (Thermo Scientific; catalog no.: AB1453B). qRT–PCR was performed according to the manual of Maxima SYBER Green/ROX qPCR Master Mix (Thermo Scientific; catalog no.: K0223). Real-time qPCR experiments were then performed. *β*-Actin was used as an endogenous reference gene for normalization of the GPD1 expression levels. The primers were as follows: mouse *GPD1* F-5′ ATACTCGGAGCCCACACCC 3′. Mouse *GPD1* R-5′ TTAGCTTTCTGCCCCCGATG 3′.

### Statistical analysis

Unless otherwise mentioned, all statistical tests were performed in GraphPad Prism 10.0. The specific statistical analysis is mentioned in each figure legend. For two-way ANOVA, all tests were performed with two-tailed analysis. Error bars are reported as SD of the mean or SEM, specified in the figure legends. All N values are provided in the figure legends.

## Data availability

Data are available by request to the corresponding author: jgalligan@pharmacy.arizona.edu.

## Supporting information

This article contains supporting information. Full list of proteins identified using SILAC can be found in Table S1, provided as an excel file (52).

## Conflict of interest

The authors declare that they have no conflicts of interest with the contents of this article.
