# Supplementary material for: Glyoxalase 1 is a proadipogenic gene
Source: J Biol Chem. 2025 Nov 7;301(12):110926. doi: 10.1016/j.jbc.2025.110926 (PMC12756636; doi:10.1016/j.jbc.2025.110926)
Supplement: Supporting Information [file mmc2.docx]

Glyoxalase 1 is a pro-adipogenic gene

Marissa N. Trujillo^1^, Wei-Chen Zhang^1^, Emely A. Hoffman^1^, Naoya Kitamura^1^, Aiden M. Phoebe^1^, James J. Galligan^1^*

^1^Department of Pharmacology and Toxicology, College of Pharmacy, University of Arizona, Tucson, AZ 85721, USA

*Corresponding author. jgalligan@pharmacy.arizona.edu

**Supplemental Tables**

**SI Table 1.** Complete list of proteins identified as differentially expressed between WT and GLO1^-/-^ cells following differentiation.

**SI Table 2.** Proteins significantly enriched in WT cells following differentiation.

**SI Table 3.** KEGG analysis of proteins identified in SI Table 2.

**SI Table 4.** Proteins significantly enriched in GLO1^-/-^ cells following differentiation.

**SI Table 5.** KEGG analysis of proteins identified in SI Table 4.

**Supporting Information and Figures**

**SI Figure S1. Generation and characterization of 3T3-L1 GLO1^-/-^ cells**

**SI Figure S2. Ablation of GLO1 prevents lipid droplet accumulation in 3T3-L1 cells**

**SI Figure S3. MGO-derived PTMs demonstrate different responses following differentiation of preadipocytes**

**SI Figure S4. Mapping of lipid binding proteins reveal alterations in fatty acid synthesis enzymes and lipid binding proteins between genotypes**

**SI Figure S5. Critical enzymes in the triglyceride and fatty acid synthesis pathway are altered following differentiation in GLO1^-/-^ cells**

**Figure S6. Quantification of glycolytic, TCA cycle and pentose phosphate pathway metabolites reveals decreased glycerol-3-phosphate levels in GLO1^-/-^ cells following differentiation.**

**Figure S7. Quantification of glycolytic, TCA cycle and pentose phosphate pathway metabolites reveals decreased glycolysis in GLO1^-/-^ cells following differentiation**

**SI Figure S8. Quantification of CoA and carnitine species in WT and GLO1^-/-^ cells following differentiation reveals global decreases in CoA and carnitines in GLO1^-/-^ cells**

**SI Figure S9. Phosphorylation of mTOR is not altered following GLO1 ablation**

**
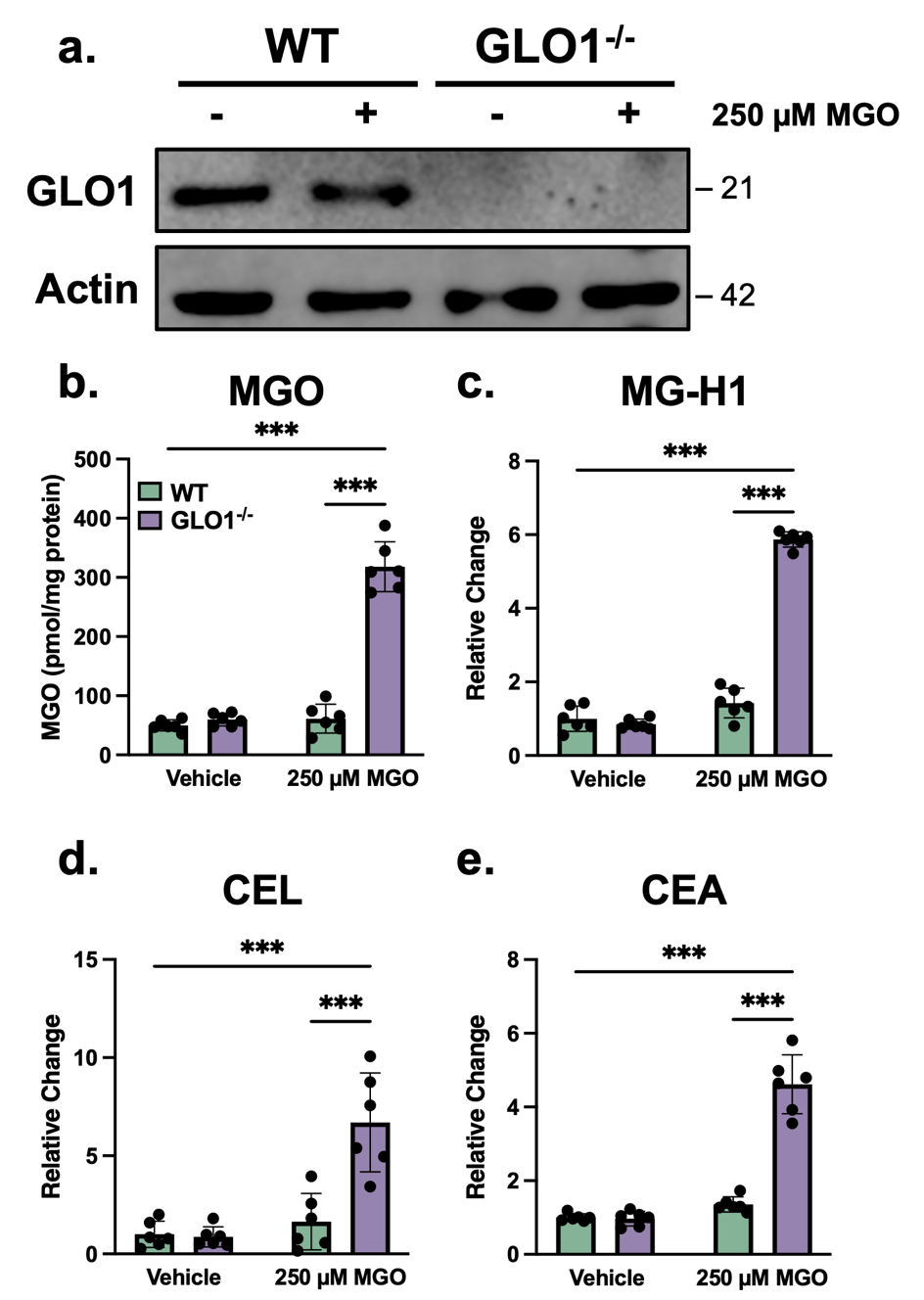
**

**SI Figure S1. Generation and characterization of 3T3-L1 GLO1^-/-^ cells. a.** Immunoblotting demonstrates GLO1 expression is undetectable, verifying knockout. **b.** Schematic for the quantification of MGO-derived PTMs. **c.** MGO levels are elevated in GLO1^-/-^ cells that have been treated with exogenous MGO. **d-f.** Quantification of MGO-derived PTMs demonstrates an increase in MG-H1, CEL and CEA in GLO1^-/-^ cells after treatment with exogenous MGO. N = 6, +/- SD. ***p < 0.001 by 2-way ANOVA.

**
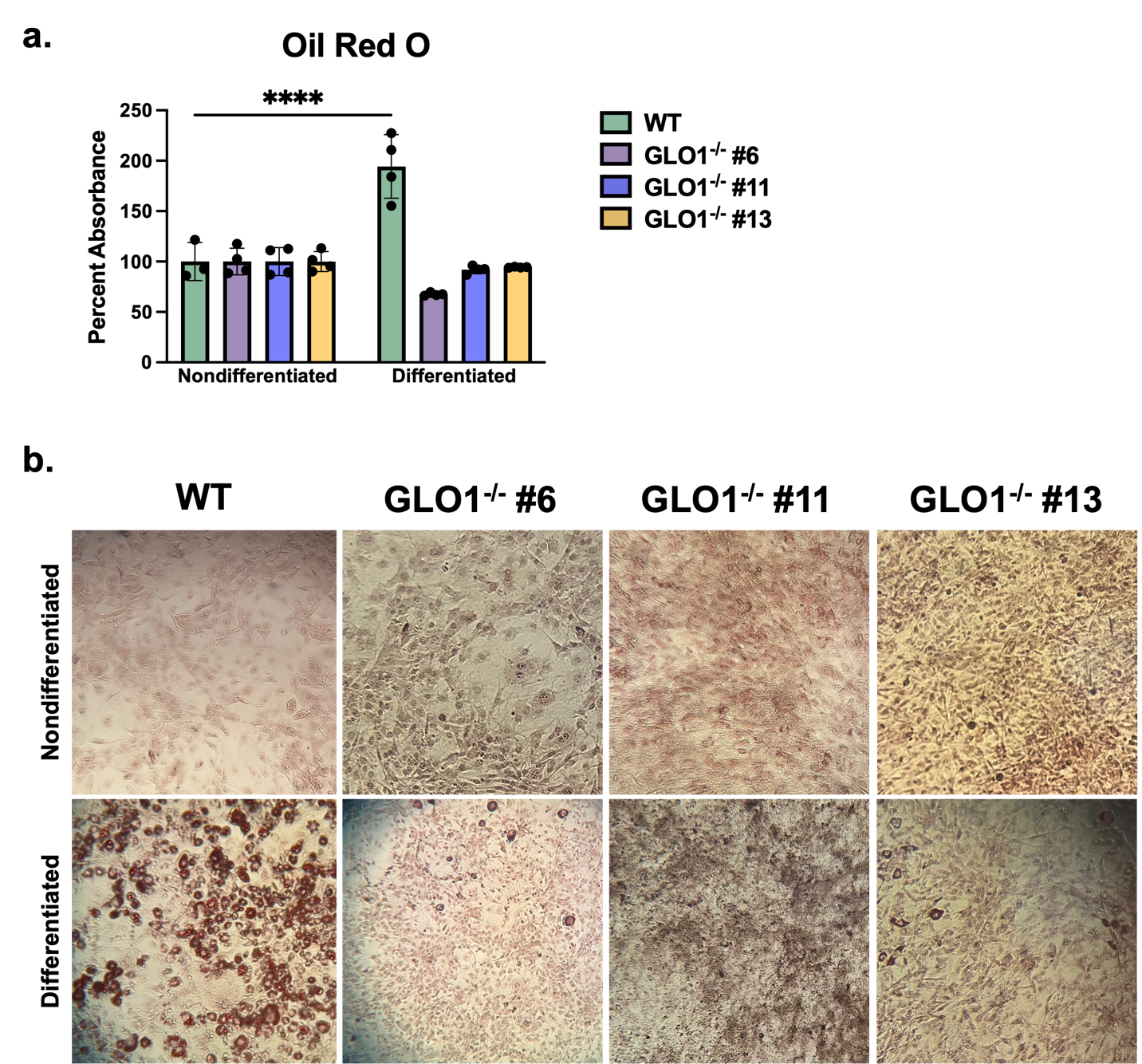
**

**SI Figure S2. Ablation of GLO1 prevents lipid droplet accumulation in 3T3-L1 cells a.** Quantification of Oil Red O, which stains for lipid droplets, demonstrates that multiple GLO1^-/-^ clones show the same phenotype, where there is a lack of lipid droplet accumulation compared to the WT differentiated cells. N = 3-4, +/- SD. ****p < 0.0001 by 2-way ANOVA. **b.** Visualization of Oil Red O staining, demonstrating little to no accumulation of lipid droplets in multiple GLO1^-/-^ clones.

**
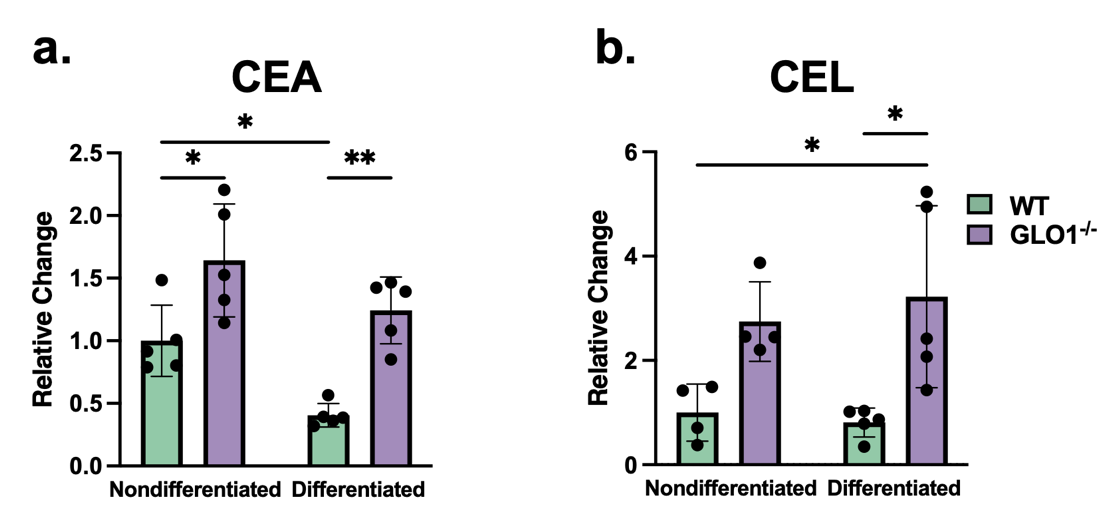
**

**SI Figure S3. MGO-derived PTMs are elevated in GLO1^-/-^ cells. a.** Quantification of CEA demonstrates increases in CEA in GLO1-/- cells with and without differentiation. **b.** Quantification of CEL reveals modest increases in GLO1^-/-^ cells following differentiation. N = 4-6, +/- SD. *p < 0.05, **p < 0.01 by 2-way ANOVA.

**
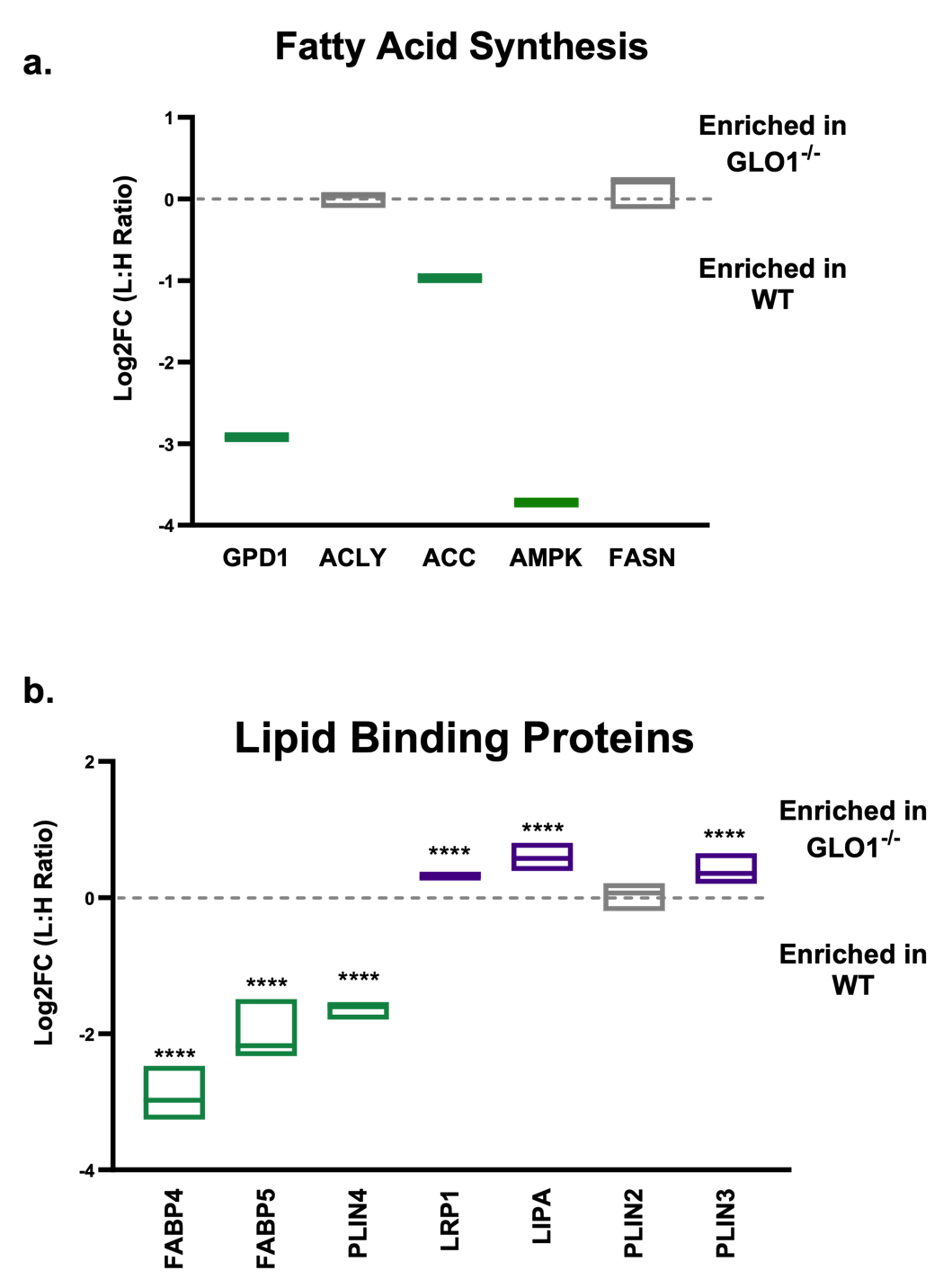
**

**SI Figure S4. Mapping of lipid binding proteins reveal alterations in fatty acid synthesis enzymes and lipid binding proteins between genotypes. a.** Mapping of fatty acid synthesis proteins reveals enrichment of GPD1, ACC and AMPK in WT cells following differentiation. ACLY and FASN expressions are unchanged. **b.** Mapping of lipid binding proteins demonstrates enrichment of fatty acid binding proteins in WT cells and enrichment of lipid binding proteins in GLO1^-/-^ cells. N = 3, +/- SD. ****p < 0.0001 by one-way ANOVA. Note: in **a**, these proteins were identified in only one data set (N = 1), thus have no statistics.

**
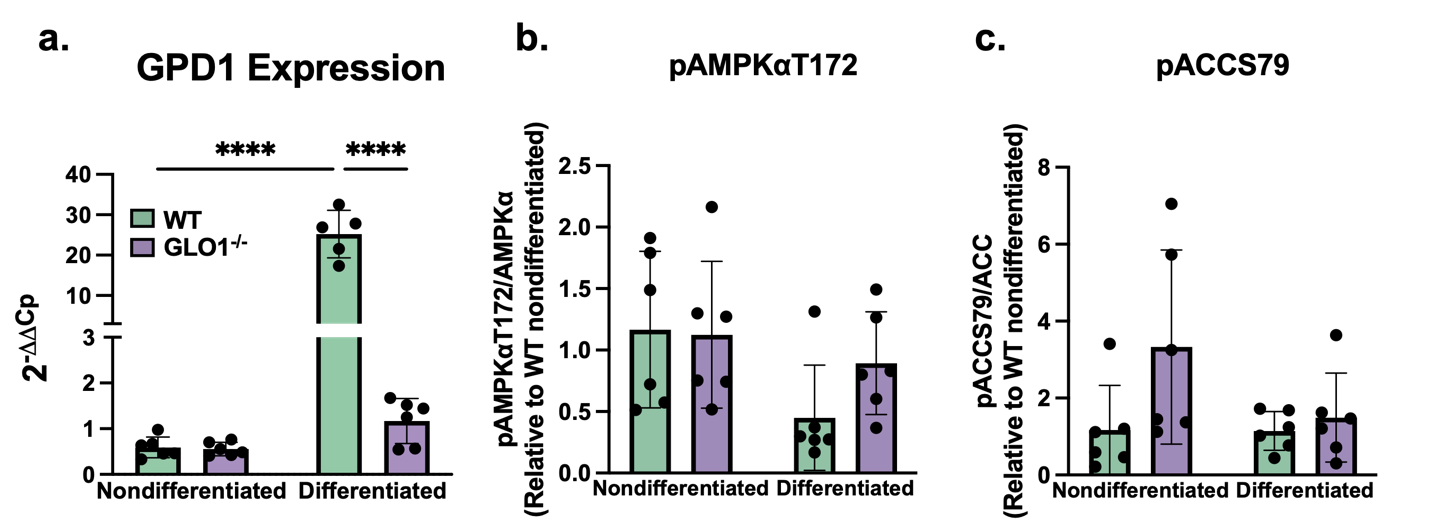
**

**SI Figure S5. Critical enzymes in the triglyceride and fatty acid synthesis pathway are altered following differentiation in GLO1^-/-^ cells. a.** Quantification of GPD1 mRNA reveals no expression of GPD1 in GLO1^-/-^ cells following differentiation, in contrast to WT cells. **b.** Quantification of immunoblotting reveals no significant alterations in neither the phosphorylation of AMPK, nor ACC phosphorylation (**c**). N = 6, +/- SD. *p < 0.05, ****p < 0.0001 by 2-way ANOVA.


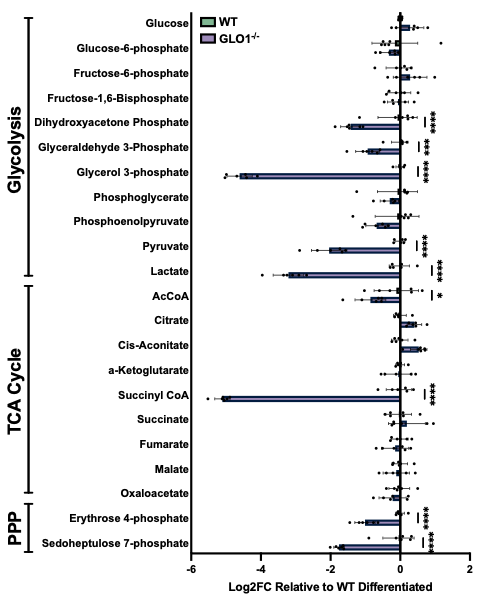


**Figure S6. Quantification of glycolytic, TCA cycle and pentose phosphate pathway metabolites reveals decreased glycerol-3-phosphate levels in GLO1^-/-^ cells following differentiation.** N = 6. *p < 0.05, ***p < 0.001, ****p < 0.0001 by 2-way ANOVA. Values are reported as Log2FC to WT differentiated.

**Figure S7. Quantification of glycolytic, TCA cycle and pentose phosphate pathway metabolites reveals decreased glycolysis in GLO1^-/-^ cells following differentiation.** N = 5-6. *p < 0.05, **p < 0.01, ***p < 0.001, ****p < 0.0001 by 2-way ANOVA. Values are reported as Log2FC to WT nondifferentiated.

**
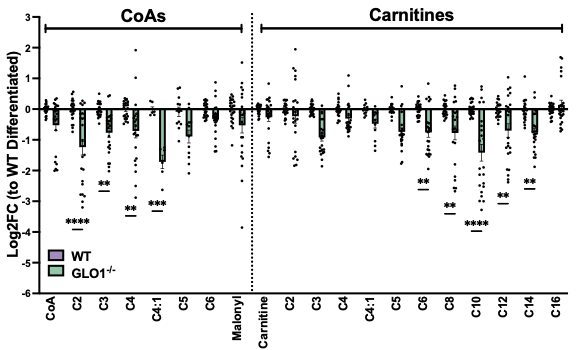
**

**SI Figure S8. Quantification of CoA and carnitine species in WT and GLO1^-/-^ cells following differentiation reveals global decreases in CoA and carnitines in GLO1^-/-^ cells.** Decreases in CoA and carnitines suggests reduced fatty acid synthesis and beta oxidation. N = 6-24, +/- SEM. **p < 0.01, ***p < 0.001, ****p < 0.0001 by two-way ANOVA. Values are reported as Log2FC to WT differentiated.


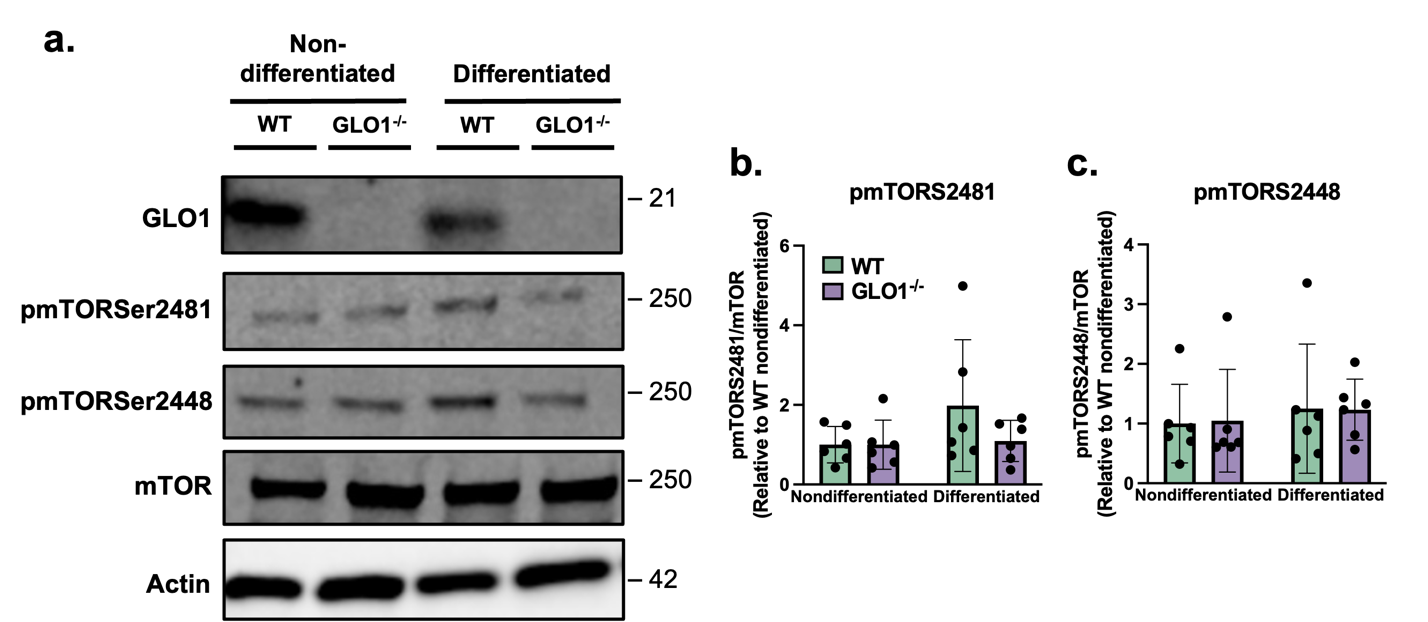


**SI Figure S9. Phosphorylation of mTOR is not altered following GLO1 ablation. a-c.** Representative immunoblotting (**a**) and quantification (**b-c**) reveals no significant differences in mTOR phosphorylation. N = 6.
